# Stenting the Unexpected: Endovascular Management of a Common Carotid Artery Pseudoaneurysm in a 79-Year-Old Woman

**DOI:** 10.7759/cureus.96288

**Published:** 2025-11-07

**Authors:** Joanna Kaszczewska, Jerzy Leszczyński, Edyta Szymanska, Piotr Kaszczewski, Krzysztof Lamparski, Michał Sajdek, Oskar Gąsiorowski, Zbigniew Gałązka

**Affiliations:** 1 Department of General, Vascular, Endocrine and Transplant Surgery, Medical University of Warsaw, Warsaw, POL; 2 2nd Department of Clinical Radiology, Medical University of Warsaw, Warsaw, POL

**Keywords:** carotid stenting, extracranial carotid artery pseudoaneurysm, false aneurysm, internal carotid artery stenosis, interventional radiology, mycotic pseudoaneurysm, pseudoaneurysm

## Abstract

Arterial pseudoaneurysms (PsA) are uncommon vascular abnormalities that may have various causes of diverse nature, and the treatment should be individualized and tailored to the patient and concomitant clinical conditions. We present a case report of a 79-year-old woman with multiple comorbidities who was diagnosed incidentally with a carotid artery pseudoaneurysm accompanied by internal carotid artery (ICA) stenosis. The pseudoaneurysm was secondary to staphylococcal sepsis, which was successfully treated three months earlier. Both the aneurysm and the stenosis were simultaneously successfully treated with an endovascular approach. Follow-up Doppler ultrasonography (DUS) performed one month later demonstrated a significant decrease in the size of the pseudoaneurysm. No postoperative complications were observed.

## Introduction

Arterial pseudoaneurysms (PsA) are rare pathologies, with an incidence rate below 1% [1], which can be either iatrogenic (vascular access complications following endovascular procedures and post-radiation aneurysms) or non-iatrogenic (secondary to trauma or infection). The risk factors of iatrogenic PsA include female sex, obesity, and anticoagulation [2].

Aneurysms in the extracranial part of the internal carotid artery (ICA), both true and false, are one of the rarest aneurysms, being the cause of 1% carotid interventions [3]. Carotid aneurysms can be both asymptomatic and symptomatic, manifesting with neurological symptoms (due to thromboembolism), enlargement of the neck with a localized, pulsating mass, or severe life-threatening bleeding secondary to lesion rupture [4].

Digital subtraction angiography (DSA) is the method of choice due to its high sensitivity and specificity, both exceeding 99%, as well as the possibility of performing simultaneous endovascular treatment [5,6].

The diagnosis of carotid artery PsA includes physical examination (pulsating neck mass) and imaging examinations such as Doppler ultrasonography (DUS) or computed tomography (CT) [7,8]. Magnetic resonance imaging (MRI) angiography is also a viable option for detecting PsA, as it is a non-invasive procedure that can help identify lesion characteristics, including dilation and the possible formation of thrombotic material [9].

## Case presentation

A 79-year-old, Caucasian woman was referred to the Vascular Surgery Department due to a newly diagnosed PsA, located on the anterior wall of the right common carotid artery (CCA), just below the carotid sinus.

The patient had a medical history of arterial hypertension, ischemic heart disease (status post-angioplasty of the left anterior descending artery with bare metal stent implantation), and type 2 diabetes mellitus. Owing to long-standing diabetes, the patient had undergone numerous ophthalmologic interventions for diabetic retinopathy and endophthalmitis. Within the month preceding the current surgery, the patient underwent a left eye vitrectomy and anterior chamber washouts with removal of inflammatory material, as well as intravitreal administration of antibiotics and antifungal agents to the right eye. The patient was on regular pharmacological treatment, including antiplatelet therapy (clopidogrel and acetylsalicylic acid), antihypertensive medications (amlodipine, bisoprolol, and torasemide), and a lipid-lowering drug (rosuvastatin).

Three months earlier, the patient had been hospitalized for sepsis caused by *Staphylococcus aureus *(*S. aureus*), originating from a urinary tract infection, which was successfully treated.

**Figure 1:**
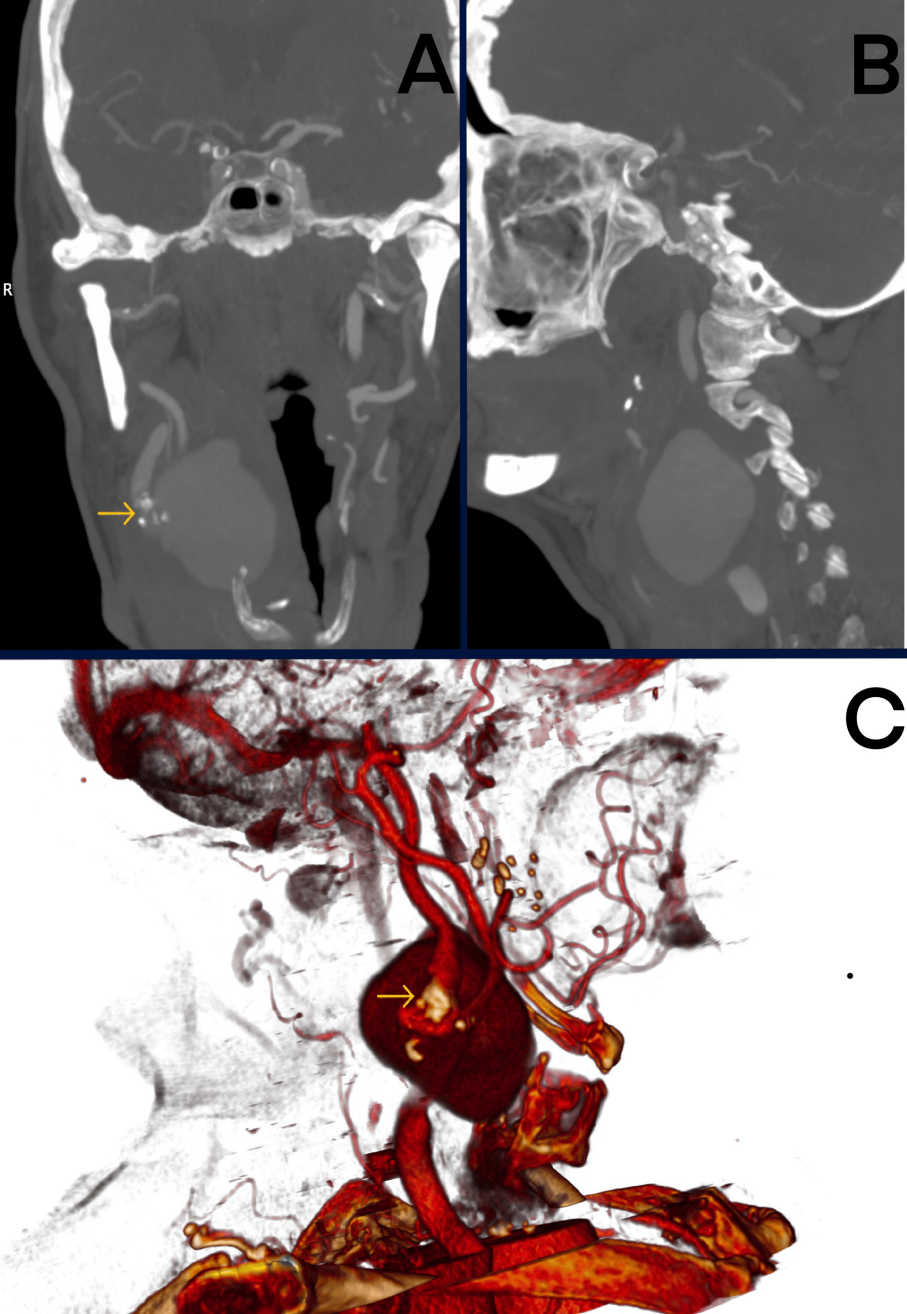
PsA of the right ICA: (A) anterior-posterior view showing the PsA, (B) right lateral view, and (C) 3D reconstruction (yellow arrows: stenosis of the ICA). PsA: arterial pseudoaneurysm, ICA: internal carotid artery

The imaging examinations confirmed the presence of the PsA, measuring 29×33×62 mm in the angio-CT. Additionally, the hemodynamically significant (>70%) stenosis of the ipsilateral ICA was diagnosed (Figure 1). Due to the rapid development and recent medical records of *S. aureus* sepsis, the mycotic etiology of the PsA was suspected.

Laboratory evaluation revealed no signs of infection, with C-reactive protein (CRP) at 1.2 mg/L, procalcitonin at 0.07 ng/mL, and white blood cell count (WBC) at 7.18×10³/µL. Blood cultures showed no bacterial growth. The patient was eligible for endovascular treatment.

**Figure 2:**
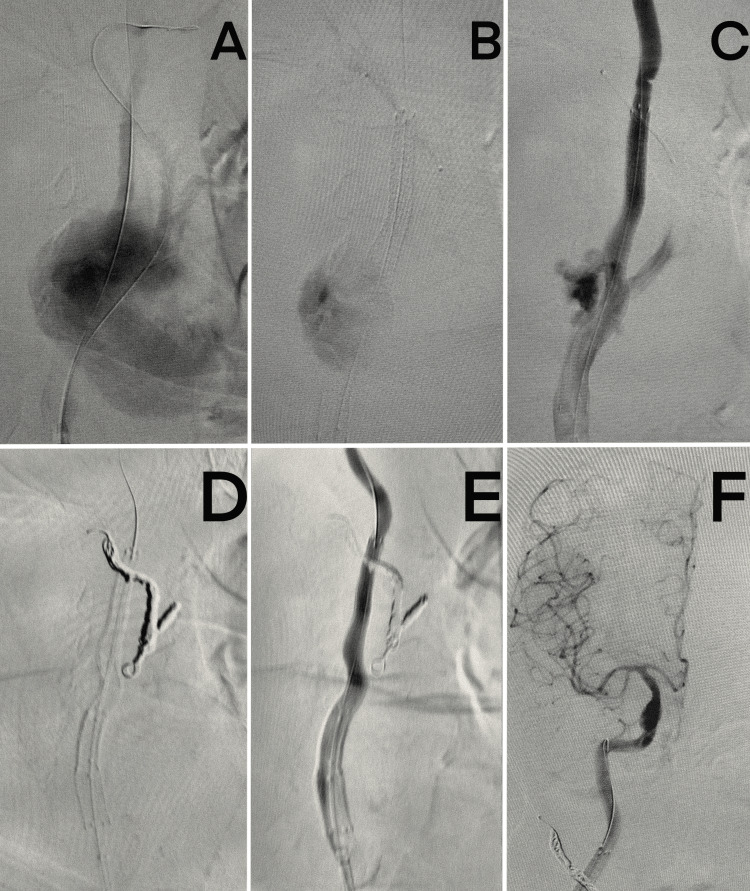
(A) Visualization of the PsA of the CCA. (B and C) Stent deployment and positioning at the site of the PsA and stenosis. (D) Coiling of the ECA. (E) Post-procedural flow: no contrast extravasation into the pseudoaneurysm; resolution of the ICA stenosis. (F) Preserved antegrade flow to the cerebral circulation. PsA: arterial pseudoaneurysm, CCA: common carotid artery, ECA: external carotid artery, ICA: internal carotid artery

From the right femoral access, the arteriography, which confirmed the presence of both pseudoaneurysm and ICA stenosis, was performed. The 7×15 mm and 5×15 Viabahn VBX Balloon Expandable Endoprosthesis (W. L. Gore & Associates, Flagstaff, AZ)stent graft was implanted to the CCA and proximal part of the ICA, respectively. Then, coil embolization of the proximal part of the external carotid artery (ECA) was performed using Ruby Coils (Penumbra Europe GmbH, Berlin, Germany). Completion angiography confirmed the complete exclusion of the pseudoaneurysm and good patency of the ICA, without stenosis (Figure 2).

The access point was sealed with the AngioSeal 8F system (Terumo, Somerset, NJ). During the procedure, the patient received 5,000 units of heparin intravenously (IV). The postoperative period was uncomplicated; control morphology and inflammatory markers were within reference range: CRP was 5 mg/dL, and WBC was 7.22×10³/µL. Hemoglobin decreased insignificantly from 9.5 to 9.4 g/dL. The patient was discharged four days following the surgery, and the control DUS was scheduled after one month.

**Figure 3:**
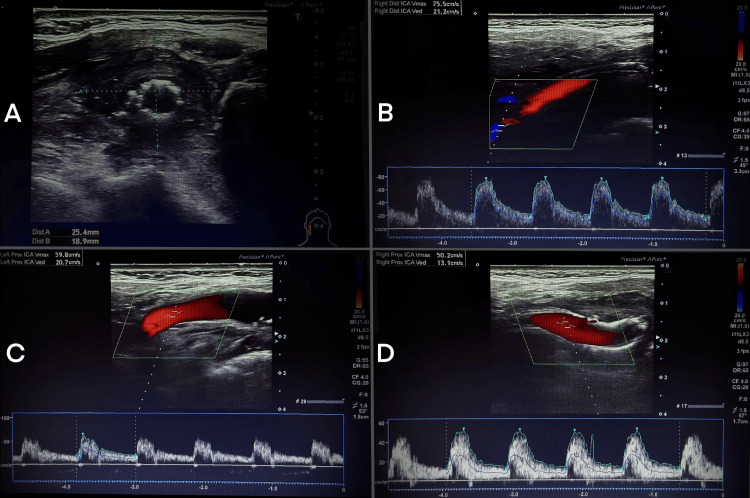
Postoperative ultrasound findings: (A) B-mode image visualizing the excluded PsA and a well-deployed stent, (B-D) triplex ultrasound (B-mode, color Doppler, and pulsed-wave Doppler) demonstrating distal flow velocity in the right ICA (B), proximal flow velocity in the left ICA (C), and proximal flow velocity in the right ICA (D). PsA: arterial pseudoaneurysm, ICA: internal carotid artery

DUS performed one month after surgery demonstrated a favorable treatment outcome. The stents were well expanded, showing normal flow velocities and no evidence of restenosis in the mid and distal segments of the CCA and in the proximal ICA. Adjacent to the distal CCA and proximal ICA, a thrombus measuring 25×19×10 mm was visualized, consistent with an excluded aneurysm without flow. No flow was detected in the right ECA (Figure 3).

## Discussion

Carotid aneurysms account for less than 1% of all aneurysms [3]. The condition is predominantly caused by trauma. Bacterial etiology is more likely to be the cause of PsA in the pediatric population [10]. In the pediatric population, PsA may also occur as a result of catheterization of the carotid artery, which is used as an access route in the treatment of congenital heart defects [11]. Mycotic aneurysms, which are most common in the aorta, are a complication of endocarditis and account for less than 3% of all aneurysms [12,13]. They can also be caused by sepsis [12]. *Staphylococcus aureus* is the most common pathogen responsible for carotid PsA. Other potential causative organisms include *Streptococcus* spp.,* Salmonella *spp.,* Klebsiella *spp., and *Escherichia coli *[14].

With the past medical history of sepsis caused by *S. aureus*, our patient had risk factors for developing PsA.

The most common clinical sign is a pulsatile mass. Additional symptoms may include neurological manifestations such as stroke, transient ischemic attack (TIA), or cranial nerve palsies [4].

The diagnostic procedure can be accelerated with the DUS, in which the “yin-yang pattern” might be observed, reflecting turbulent blood flow within the pathological vessel. In CT imaging, the yin-yang pattern arises from incomplete opacification of the vessel lumen, with one portion filled with contrast material and the other showing reduced blood flow [15].

Both CT and MRI demonstrate a contrast-enhancing wall and surrounding inflammatory tissue in the diagnostic process of a mycotic aneurysm. Gadolinium-enhanced MRI can reveal inflammatory processes in the aneurysm wall [9], while thrombus enhancement reflects organization and neovascularization [16]. Contrast enhancement on CT likewise indicates inflammation, and both modalities demonstrate enhancing aneurysm walls with surrounding inflammatory tissue, essential for diagnosing mycotic aneurysms [17]. The gold standard for diagnosis is DSA [5].

In 2009, Attigah et al. proposed a classification of both true and false extracranial carotid artery aneurysms based on CT or DSA, defining five types according to their anatomical location: type I, isolated aneurysm of the ICA; type II, aneurysm of the ICA including the bifurcation; type III, aneurysm at the carotid bifurcation; type IV, aneurysm involving both the ICA and the CCA; and type V, isolated aneurysm of the CCA. According to this classification, our patient had a type V lesion [18].

The management of PsA of the ICA includes both pharmacological and surgical treatment (open surgery, endovascular procedure, and hybrid approach). The conservative approach is preferred for asymptomatic patients with stable lesions as a prevention of embolic strokes and typically involves antiplatelet or anticoagulation therapy [19].

Filo et al. suggest that lesion diameter ≤ 6 mm and patient age ≤ 50 years may be useful predictors of PsA resolution with pharmacological treatment only [20]. In the Cervical Artery Dissection in Stroke Study (CADISS) trial, patients placed on either single or dual antiplatelet therapy were compared with those on anticoagulants [21]. Similarly, the aspirin versus anticoagulation in cervical artery dissection (TREAT-CAD) study compared patients placed on aspirin versus warfarin. In both trials, aspirin was shown to be noninferior to anticoagulant therapy [22]. It is worth mentioning that warfarin was used as an anticoagulant because the CADISS trial started before novel oral anticoagulants (NOACs) [21]. Therefore, to our knowledge, there is a lack of similar trials regarding NOACs, although they are generally thought to be safer and easier to monitor.

A study by Fankhauser et al. proves that symptomatic aneurysms are more frequent, accounting for 52% of all cases. The proposed surgical treatment includes open surgical treatment, with the usage of synthetic and natural patches (Dacron, saphenous vein, or bovine pericardium) and endovascular treatment, which was used more often [19].

The open surgical treatment of carotid aneurysms is done in a similar protocol to carotid endarterectomy. Five techniques for open surgical intervention, identified by Garg et al., include aneurysm clipping, excision with primary anastomosis, excision with interposition grafting, extracranial-to-intracranial bypass, and carotid artery ligation [23].

Xu et al. recently demonstrated that hybrid and endovascular approaches effectively treat both true and false aneurysms, with a reported success rate of 90% [24].

Although open surgical treatment is a relatively effective option, it carries a high risk of cranial nerve injury; therefore, endovascular treatment should be considered as a safer alternative [25]. In our patient, an endovascular approach with a VBX Stent Graft was performed, resulting in both clinical and technical success.

The endovascular treatment of PsA can be done with different techniques, which include coiling, bare metal stent, and stent graft. If the treatment of a carotid PsA requires permanent occlusion of the ICA, a balloon test occlusion (BTO) is necessary to assess whether there is adequate collateral circulation. If the results indicate insufficient collateral flow, the chosen treatment method may need to be altered. Angiography, with evaluation of both the anterior cerebral artery (ACA) and the middle cerebral artery (MCA), can help predict the outcome of the BTO [26].

Kumar et al. reported that parent artery occlusion (PAO) was one of the most common procedures (10/20), followed by arterial stenting (7/20) [27].

The use of covered stents allows for patency of the artery; therefore, it is the treatment of choice. Although bare metal stents are easily deployed, they have a risk of recanalization of the PSA. Stent grafts allow for rapid obliteration of the PSA, although their deployment is tougher [28].

Flow-diverter stents (FDS) must be considered in the treatment of carotid aneurysms. Wang et al. emphasized their potential value in treating lesions of the distal segment of the extracranial ICA [29]. In a series by Cinar et al. involving 19 patients with extracranial carotid aneurysms, FDS implantation was technically successful in all cases, with complete symptom resolution in those who were symptomatic [30]. Beaty et al. also reported a case of Horner’s syndrome due to a carotid PsA successfully treated with an FDS, leading to full clinical recovery [31]. Despite these promising outcomes, the use of FDS in extracranial carotid pathology remains relatively novel, and further studies are required to define its long-term efficacy and safety.

As stated in the CIRSE guidelines, liquid embolic agents are especially advantageous for embolization of distal, small, or tortuous arterial branches, where coil deployment is technically difficult or impractical due to vessel anatomy [32]. According to the American Heart Association (AHA) and American Stroke Association (ASA) guidelines, coil embolization and surgical clipping are the primary treatments for aneurysmal subarachnoid hemorrhage; flow diverters may be considered in select cases but are not routinely used in the acute setting [33]. Moreover, it is pointed out that in situations involving high-flow vessels, the embolic material may migrate [34]. Considering that the carotid arteries are high-flow vessels and, as highlighted in the previously cited studies, liquid embolic agents are less frequently employed in this region, the patient underwent coil embolization.

Although endovascular techniques have lower morbidity, they are not free of complications. Postoperative complications of endovascular techniques may arise during or after the treatment. The possible complications include arterial dissection, embolic stroke or TIA, occlusion, late stenosis, or endoleak [35]. If the patient had focal symptoms of discomfort prior to the surgery, they might not subside after stent-graft implantation [36].

Mycotic PsA are usually treated via open surgical excision due to the possibility of debridement of the tissues. Endovascular treatment might be an option when the simultaneous antibiotic treatment was introduced and when the patient is considered high-risk for open surgery [37-39].

According to the study by Luo et al., implementation of preoperative four-week antibiotic treatment, based on the culture results and sensitivity testing, with simultaneous leukocyte count and temperature monitoring, followed by postoperative six-week antibiotic therapy, may reduce the risk of premature death due to fatal infectious complications [40].

Lui et al. report mycotic PsA secondary to *Mycobacterium tuberculosis *infection, which was treated successfully with antimicrobial therapy and open surgical excision with end-to-end anastomosis between the CCA and ICA [41].

Deiana et al. report a mycotic PsA located in the carotid bifurcation. The symptoms included fever, elevated white blood cell count, and a simultaneous pulsating painless mass. After preliminary antibiotic treatment, open surgical treatment with vein graft reconstruction was conducted [37].

Marianna et al. describe the formation of a PsA linked to such staphylococcal abscesses. After preliminary stabilization, which included implantation of a covered stent in the CCA and ICA, a surgical excision of the PsA was performed, which included the removal of the stent and restoration of the continuity of the vessel with the great saphenous vein [38].

Raso et al. describe a patient with neurological symptoms caused by ICA stenosis: left-sided hemiparesis and dysarthria. Two weeks after the stent implantation, he presented with swelling in the groin puncture and swelling near the right shoulder, with positive blood cultures for *S. aureus*. After antibiotic treatment, the left-sided pulsating mass was reported. The patient underwent open surgical excision of PsA, located near the stenting area, with ligation of the ECA. Continuity of the vessel was accomplished with the saphenous vein graft [42].

Sano et al. present a case of successful endovascular treatment of an ICA aneurysm caused by *Aspergillus* sinusitis. Due to a previous history of hemispheric ischemia, the patient was disqualified from coiling; however, the possible treatment was a combination of stent graft, which allowed the continuous flow in the ICA, and coiling of the aneurysm [43].

The presented case report of a 79-year-old woman diagnosed with a PsA of the CCA showcases that an incidental diagnosis and later endovascular treatment might prevent potentially life-threatening complications. The patient underwent successful stent-graft implantation with simultaneous treatment of ICA stenosis and coil embolization of the ECA. The procedure yielded favorable short- and long-term outcomes.

## Conclusions

This case highlights the importance of selecting an appropriate intervention strategy in the management of carotid artery PsA, especially those incidentally detected. Even in complex clinical scenarios, such as older patients with multiple comorbidities and a history of prior infections, this rare and severe condition, mycotic PSA, can be successfully treated using a minimally invasive endovascular approach, while simultaneously addressing any stenosis.

Combining stent-graft implantation with coil embolization of the external carotid artery can provide durable exclusion of the PsA while restoring adequate blood flow and resolving the stenosis, thereby minimizing the risk of rupture or neurological complications. Ultimately, this case reinforces the value of a multidisciplinary strategy and individualized intervention to achieve optimal patient outcomes. Stent-graft implantation addresses both issues simultaneously, potentially providing a minimally invasive endovascular-only solution.
